# Severe falciparum malaria complicated by prolonged haemolysis and rhinomaxillary mucormycosis after parasite clearance: a case report

**DOI:** 10.1186/s12879-015-1285-1

**Published:** 2015-12-03

**Authors:** Katherine Plewes, Richard J. Maude, Aniruddha Ghose, Arjen M. Dondorp

**Affiliations:** Mahidol Oxford Tropical Medicine Research Unit, Faculty of Tropical Medicine, Mahidol University, 420/6 Rajvithi Road, Rajthevee, Bangkok 10400 Thailand; Centre for Tropical Medicine and Global Health, Nuffield Department of Medicine, University of Oxford, Oxford, United Kingdom; Department of Medicine, Chittagong Medical College Hospital, Chittagong, Bangladesh

**Keywords:** Falciparum malaria, Prolonged haemolysis, Mucormycosis

## Abstract

**Background:**

Severe falciparum malaria may be complicated by prolonged haemolysis and recurrent fever after parasite clearance. However, their respective etiologies are unclear and challenging to diagnose. We report the first case of severe falciparum malaria followed by prolonged haemolytic anaemia and rhinomaxillary mucormycosis in a previously healthy adult male.

**Case presentation:**

A 30-year old Bangladeshi man was admitted with severe falciparum malaria complicated by hyperlactataemia and haemoglobinuria. Prior to admission he was treated with intravenous quinine and upon admission received intravenous artesunate and empiric ceftriaxone. Thirty hours later the peripheral parasitaemia cleared with resolution of fever and haemoglobinuria. Despite parasite clearance, on day 3 the patient developed recurrent fever and acute haemolytic anaemia requiring seven blood transfusions over six days with no improvement of his haemoglobin or haemoglobinuria. On day 10, he was treated with high-dose dexamethasone and meropenem with discontinuation of the ceftriaxone. Two days later the haemoglobinuria resolved. Ceftriaxone-induced haemolysis was the suspected final diagnosis. On day 16, the patient had progressively worsening right-sided facial pain and swelling; a necrotic ulceration of the hard palate was observed. Rhinomaxillary mucormycosis was diagnosed supported by microscopy findings. The patient initially responded to treatment with urgent surgical debridement, itraconazole, followed by two weeks of amphotericin B deoxycholate, however was subsequently lost to follow up.

**Conclusions:**

This case highlights the range of potential alternative aetiologies of acute, prolonged haemolysis and recurrent fever following parasite clearance in severe falciparum malaria. It emphasizes the importance of a high degree of suspicion for alternative causes of haemolysis in order to avoid unnecessary treatments, including blood transfusion and steroids. It is critical to consider and identify common invasive bacterial and rare opportunistic co-infections as a cause of fever in severe malaria patients remaining febrile after parasite clearance to promote antimicrobial stewardship and prompt emergency care.

## Background

The mortality rate of adult severe malaria remains high at between 10 % and 30 %. In surviving patients recovery is usually complete, and neurological sequelae occur in less than 1 % [1]. Rare complications following severe falciparum malaria include acute, prolonged haemolytic anaemia and invasive fungal infections [2, 3]. Prolonged anaemia following parasite clearance in falciparum malaria is multifactorial. Proposed mechanisms include hypoproliferative erythropoiesis, ineffective erythropoiesis, red blood cell (RBC) membrane alteration with reduced survival, and continued peripheral haemolysis [2]. Broadly, the causes of ongoing haemolysis after parasite clearance are complex and include: (1) immune-mediated, secondary to parasite antigens, complement activation, splenic retention, or drug-induced autoimmune haemolysis (i.e. aryl-amino-alcohol containing antimalarials) [4–7], and (2) non-immune mediated, secondary to rupture of sequestered schizonts and uninfected RBCs and oxidative stress [8, 9]. Recently, delayed onset haemolysis after falciparum malaria has been associated with parenteral artesunate use in non-immune patients with high ring stage admission parasitaemias [10–19]. Three patterns of post-treatment anaemia following malaria have been proposed namely, ‘rising’, ‘persistent’ and ‘post-artesunate delayed haemolysis’ [20].

Invasive fungal infections complicating severe falciparum malaria infections are rare, particularly mucormycosis [21–27]. Zygomycetes can cause life-threatening angioinvasive mucormycoses that present as rhinocerebral, pulmonary, renal, gastrointestinal, cutaneous and/or disseminated infection. Individuals with an immunocompromising condition or iron overload are particularly predisposed to these acute-onset, rapidly progressive and aggressive infections. The overall mortality is 44 %, however in the case of haematogenous disseminated disease up to 94 % of patients have a fatal outcome [28]. Successful management of mucormycosis requires prompt diagnosis, reversal of underlying immunosuppression, and urgent surgical debridement together with anti-fungal therapy. Only one case of disseminated mucormycosis complicating severe falciparum malaria has been reported [3]. We report the first case of rhino-maxillary mucormycosis following *Plasmodium falciparum* infection complicated by prolonged haemolysis after parasite clearance.

## Case presentation

A 30-year-old previously well Bangladeshi male was admitted to a local tertiary care hospital with a 20-day history of fever, chills, rigors, headache, myalgias, and anorexia. He had been suffering from nausea, vomiting, diarrhoea, jaundice with scleral icterus and dark urine for five days. One day prior to admission, he was diagnosed with malaria on thick smear at a private hospital, however parasite density result was unavailable. There he was treated with intravenous quinine (750 mg 8-hourly x five doses) and azithromycin, due to limited availability of first line therapy of artesunate, before being referred for further management.

Past medical history was significant for three prior malaria infections. His most recent episode of suspected vivax malaria was 30 days prior to admission, which was partially treated with an incomplete course of chloroquine. He had no history of jaundice, liver disease, transfusions, malignancy, or autoimmune disease. There was no family history of jaundice or iron treatments. He did not take any regular or traditional medicines. There was no history of betel nut consumption.

On admission, he had a temperature of 39.7 ^o^C, Glasgow Coma Score (GCS) 15/15, blood pressure (BP) 127/60 mmHg, heart rate (HR) 127 beats per minute, a respiratory rate (RR) of 24 breaths per minute with oxygen saturation (SaO_2_) of 93 % on room air, and body weight of 65 kg. He appeared unwell with pronounced scleral icterus, black urine, hepatomegaly and splenomegaly (4 cm and 5 cm below the costal margin, respectively). There was no conjunctival suffusion or lymphadenopathy.

Admission peripheral blood smear confirmed a diagnosis of falciparum malaria (360 parasites/μl). Severity criteria included hyperlactataemia (lactate 6.8 mmol/L), hyperbilirubinaemia (total bilirubin 58 μmol/L), and 4+ haemoglobinuria (blackwater fever); hence the case was classified as severe malaria (Table 1). He was promptly treated with intravenous artesunate 120 mg, 2.4 mg/kg body weight (Guilin Pharmaceuticals, China) at 0, 12, 24 and 48 h (four doses), in addition to empiric ceftriaxone 2,000 mg every 12 h, as per local hospital practice. After 30 h his peripheral parasites cleared with resolution of the fever and haemoglobinuria, and he was switched to oral artemether/lumefantrine (AL) combination 80/480 mg (Novartis, Switzerland).

**Table 1:** Laboratory investigations

Parameter	Day 0	Day 3	Day 4	Day 7	Day 10	Day 28/29	Normal Range
Haemoblobin (g/L)	107	32	31	36	34	68	140–180
Haematocrit (%)	28.6	10	8.4	10	--	20.4	47–54
Mean corpuscule volume (fL)	79.4	--	76.4	73.5	--	83	76–96
Platelets (x10^3^/μl)	38	--	32	160	180	160	150–400
White blood cells (x10^3^/μl)	8.4	3.2	3.2	4.5	8	3.8	4–10
Lactate (mmol/L)	6.8	3.6	--	--	--	--	0.9–1.7
Creatinine (μmol/L)	185	177	159	133	265	133	35-124
Bicarbonate (mmol/L)	18.3	17.1	--	29.6	--	23.2	23–28
Base excess (mmol/L)	−7	−8	--	0	--	0	(-2)–(+3)
Anion gap (mmol/L)	19	17	--	15	--	--	10–20
Total bilirubin (μmol/L)	58	82	75	--	--	--	3–21
Indirect bilirubin (μmol/L)	34	--	53	--	--	--	2–14
Alanine aminotransferase (IU/L)	23	59	--	--	1260	57	5–41
Alkaline phosphatase (IU/L)	66	--	74	--	--	--	44–147
Gamma-glutamyl transferase (IU/L)	18	--	13	--	--	--	<55
Lactate dehydrogenase (IU/L)	--	--	2950	--	--	--	140–280
International normalization ratio	--	1.3	--	--	2.18	--	0.8–1.2
Partial thromboplastin time (seconds)	--	15	--	--	27	--	<30
Erythrocyte sedimentation rate (mm)^a^	--	155	--	147	160	61	0–10

^a^Westergren method

**Fig. 1:**
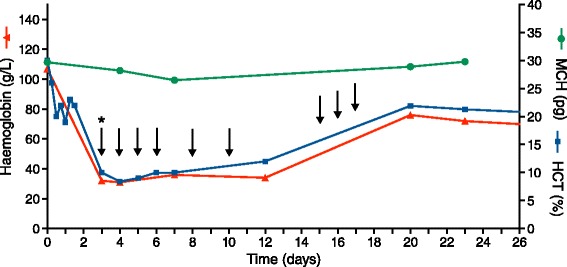
Haemoglobin, haematocrit (HCT) and mean corpuscular haemoglobin (MCH) profile during hospital admission. Arrows indicate timing of one-unit whole blood transfusions. Asterisk indicates initial transfusion of 3 units

On the third day of admission he developed fever, confusion, dyspnoea (RR 28, SaO_2_ 90 %), tachycardia (HR 106), hypotension (BP 100/80 mmHg), recurrent 4+ haemoglobinuria with severe microcytic anaemia (haemoglobin of 32 g/L) accompanied by elevated lactate dehydrogenase and bilirubin, consistent with acute haemolytic anaemia (Table 1, Fig. 1). Pre-transfusion peripheral blood smear showed no schistocytes and reticulocytes of 2 % (reticulocyte production index 0.15 (compared to RPI of 0.3 on day 3 in blackwater fever patients [29]). Direct antiglobulin test (DAT; anti-IgG and anti-C3d) was negative and indirect antiglobulin test (IAT) was positive. Glucose was normal. Blood and urine bacterial cultures were negative. Glucose-6-phosphate dehydrogenase testing and haemoglobin typing were not available. Human immunodeficiency virus, syphilis, hepatitis A, hepatitis B and hepatitis C serology were negative. Hepatitis B antigen was negative. He promptly received transfusion of three units matched whole blood. Intravenous artesunate was substituted for AL despite parasite clearance due to the notion that the recurrent fever must be due to malaria. In total, he received seven doses of artesunate (total dose of 840 mg) and three doses of AL (total dose of artemether 240 mg, and lumefantrine 1,440 mg). Empiric amikacin 500 mg, 7.5 mg/kg 8-hourly was added on day 6 to broaden antibacterial coverage for potential multi-drug resistant gram-negative bacteria given his ongoing fever despite negative blood and urine cultures. The haemolysis continued for nine days and he required a total of 10 transfusions (Fig. 1).

On day 10, bone marrow biopsy revealed gross hypercellularity with erythroid hyperplasia and no evidence of lymphoma, leukaemia, or haemophagocytic syndrome. Meropenem (500 mg 8-hourly) was empirically substituted for amikacin and ceftriaxone given his renal dysfunction to cover potential extended-spectrum β-lactamase producers, and dexamethasone 5 mg 6-hourly was initiated for suspected autoimmune haemolytic anaemia. The haemoglobinuria resolved two days after steroid treatment and ceftriaxone discontinuation. His haemoglobin slowly recovered, reaching 76 g/L by day 20.

**Fig. 2:**
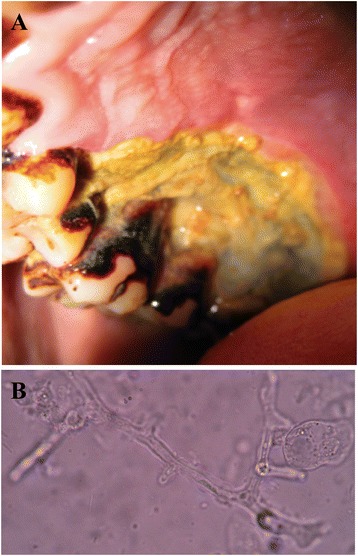
Oral ulceration. **a** Ulcer on right upper hard palate measuring 3x2cm, covered with yellow slough and a posterior area of necrosis. The borders of the ulcer were undermined. **b** Light microscopy x100 image of wet mount from swab sample of hard palate ulceration showing long, broad, aseptate hyphal elements displaying 90° branching consistent with *Mucor*

**Fig. 3:**
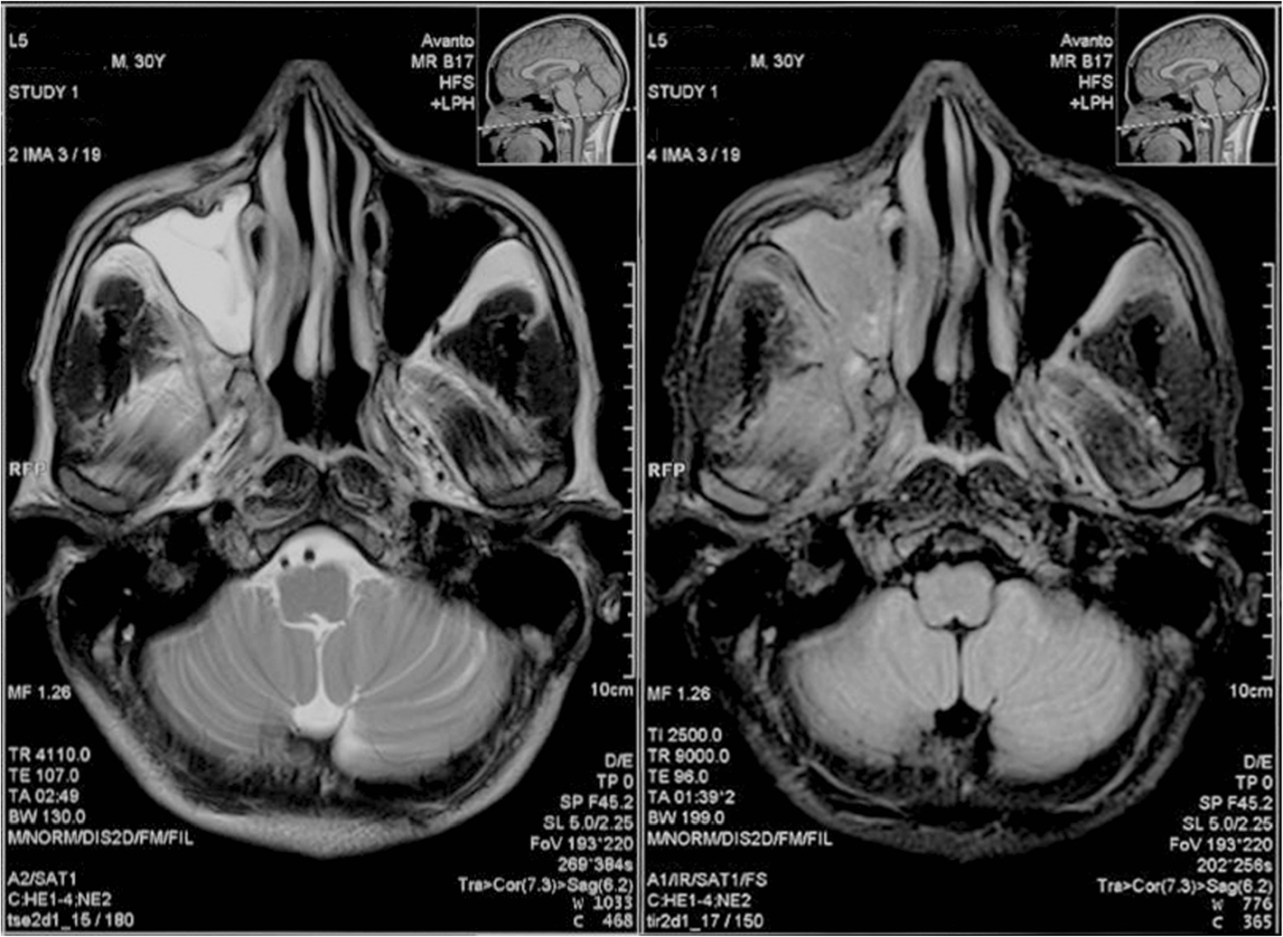
MRI brain. Opacification and mucosal thickening of the right maxillary and ethmoid sinuses. Left – T2 weighted image; right– T1 weighted image

On day 16, he had ongoing fever with right-sided facial pain and swelling. A large, 3 × 2 cm ulcer on the right upper hard palate covered with yellow slough and an area of necrosis was observed (Fig. 2a). The right maxillary sinus was tender on palpation. He had pain with right eye extra-ocular movements but no ocular discharge, proptosis, pupil asymmetry, diplopia, or decreased visual acuity. There was no nasal or ear involvement. A swab of the hard palate ulceration revealed long, broad, aseptate hyphal elements displaying 90° branching consistent with *Mucor*, (Fig. 2b). Fungal cultures failed to grow. MRI showed extensive right-sided opacification and mucosal thickening of the maxillary and ethmoid sinuses with no radiographic evidence of orbital or cerebral extension (Fig. 3). He underwent prompt surgical debridement of the oral ulcer and right maxillary sinus. His steroids were discontinued and he was started on oral itraconazole 100 mg 12-hourly until first line treatment with amphotericin B became available nine days later. Intravenous amphotericin B deoxycholate 50 mg daily, was continued for 14 days with improvement of his facial swelling and oral ulceration. The patient was pleased with his clinical improvement and hospital care, however due to a family emergency in his remote village he left hospital against medical advice and could not attend follow up.

## Conclusions

The mechanism of the haemolytic component of anaemia following parasite clearance in falciparum malaria is poorly understood and challenging to differentiate. This case does not completely conform to one of the three patterns of post-treatment malaria anaemia as it appeared bimodal within a short time period, where the initial episode of haemolysis occurring while parasitaemic resolved by 30 h after artesunate treatment, and a second episode of massive haemolysis began on day 3 with 75 % drop in haemoglobin that persisted for 10 days requiring multiple transfusions. This case highlights that drug-induced haemolysis may contribute. Several antimalarial drugs can cause haemolysis by differing mechanisms including quinine, mefloquine, halofantrine, and primaquine. Two possible culprit drugs in our case include quinine and ceftriaxone. Quinine was given prior to admission, and blackwater fever (intravascular haemolysis with haemoglobinuria) was observed on his initial presentation with *P. falciparum* parasitaemia. Quinine-induced haemolytic anaemia is considered drug-dependent and proposed to occur through an immune-complex mechanism [30, 31], however since the acute haemolysis began on day 3, by which time around 95 % of the drug would have been eliminated [32], and the DAT was negative, quinine seems less likely to be the cause. Drug-induced immune haemolytic anaemia (DIIHA) is rare but the commonly cited incidence of 1 per million people is likely an underestimate due to under-diagnosis [33]. In patients with DIIHA, ceftriaxone is the second most common antimicrobial cause after cefotetan [30, 33]. The onset of ceftriaxone-induced DIIHA ranges from 30 min to 34 days following exposure [30]. In this case, the negative DAT and positive IAT suggest the presence of an alloantibody or free autoantibody. The DAT is typically positive in most cases of drug-induced haemolytic anaemia but a negative DAT is possible if massive haemolysis occurs before testing [33, 34]. A diagnosis of ceftriaxone-induced DIIHA is supported in our case because the haemoglobinuria stopped two days after discontinuing ceftriaxone, however the haemoglobin was still recovering to baseline 2 weeks post-ceftriaxone discontinuation. Steroids do not improve DIIHA, but were administered in this case for potential autoimmune haemolytic anaemia [33]. Since steroid was started at the same time that ceftriaxone was stopped, establishing the cause of haemolysis is confounded. The diagnosis of ceftriaxone-induced haemolytic anaemia could not be confirmed because specific ceftrixone- antibody testing was not available.

The differential diagnosis for acute onset haemolytic anaemia with positive IAT and negative DAT following parasite clearance includes alloantibody mediated from previous transfusion, drug-induced haemolytic anaemia, microangiopathic haemolytic anaemia, an intrinsic RBC abnormality, or may be a non-specific finding [30, 33]. Delayed haemolytic transfusion reactions are generally mild and the onset of haemolysis is usually 2 to 11 days after subsequent transfusion. This diagnosis was unlikely as there was no history of previous blood transfusion and haemolysis started before the transfusions. Reports of post artesunate delayed haemolysis typically occur in hyperparasitaemic patients and manifest 7–14 days following the first dose of artesunate [19, 20]. This diagnosis is unlikely since this patient did not have a high parasitaemia and haemolysis started three days after the start of artesunate. The available blood results do not support the diagnosis of a microangiopathic haemolytic anaemia, intrinsic RBC abnormality or functional asplenia since there were no obvious red cell abnormalities or Howell-Jolly bodies on peripheral blood smear.

Fever in severe malaria usually resolves by day 4 to 5 [35]. Thus, recurrent or prolonged fever after severe malaria should prompt investigation into alternative causes. We identified a necrotic oral ulcer, from which a sample showed *Mucor* morphology on slide microscopy (Fig. 2). While our diagnosis was not confirmed by culture, this is not a prerequisite for the diagnosis since these organisms are difficult to grow and frequently cultures yield no growth. While mucormycosis is frequently fatal in immunocompromised hosts, such as those with haematological malignancies and diabetes mellitus, cases in patients without any immune impairment have been reported [28, 36]. These infections manifest as localized cutaneous or disseminated deep infections and are characterised by necrotic lesions due to the angioinvasive behavior of these organisms. Rhinocerebral mucormycosis is the most common clinical presentation of mucormycosis, representing around 60 % of all cases [37]. This category is further divided into 2 subtypes: the highly aggressive, often fatal rhino-ocular-cerebral form and the less fatal rhino-maxillary form. The latter subtype manifests as necrosis of the palate due to angioinvasion and thrombosis of the sphenopalatine and greater palatine arteries [38, 39].

Invasive fungal infections are rarely reported in association with severe malaria [22]. A recent review summarized nine cases of invasive fungal infections complicating falciparum malaria; 5 Aspergillus, 1 hyalohyphomycosis, 2 *Cryptococcus*, 1 *Absidia,* and 1 disseminated candidiasis [21–27]. The immune suppression due to malaria infection itself may predispose individuals to invasive fungal infection [3, 22]. Parasitic digestive vacuoles containing malaria pigment phagocytosed rapidly by polymorphonuclear granulocytes can cause functional exhaustion, which blunts microbicidal activity of granulocytes [40]. Neutrophil dysfunction might result from haemolysis-induced haem oxygenase-1 induction [41]. High-dose steroids may also have inhibited the polymorphonuclear leukocyte response and decreased granulocyte phagocytosis. In this case, the iron overload state secondary to massive haemolysis and multiple transfusions may have promoted fungal growth [37, 42, 43]. All reported Aspergillus and Absidia infections complicating severe malaria have occurred in patients with marked haemolysis [3, 23, 24, 26].

In this case it was difficult to definitively exclude a predisposing immunocompromising condition. However, bone marrow biopsy did not suggest an underlying haematologic malignancy, HIV serology was negative, and random glucose was normal in the setting of acute illness, although haemoglobin A1C testing was unavailable to definitely rule out diabetes. It is likely that malaria related immune suppression augmented by the administration of steroids and multiple blood transfusions with massive haemolysis (iron overload) contributed. The initial low parasitaemia is consistent with post-treatment presentation and the severity criteria present on admission are consistent with a delayed time to recovery in severe malaria patients from low transmission settings [1]. Concomitant bacterial infection was not definitively excluded since admission blood culture results were not available, however the day 0 white blood cell count was normal. The kidney and liver dysfunction that developed when mucormycosis was diagnosed has several explanations but is unlikely related to disseminated *Mucor*. More plausibly, the kidney injury was due to high cell-free haemoglobin generated during massive haemolysis, ischaemia, or nephrotoxic drugs (i.e. amikacin). The liver dysfunction was likely due to ischaemia during hypotensive episodes.

In addition to urgent surgical debridement, the recommended first line anti-fungal therapy for the treatment for mucormycosis is amphotericin B deoxycholate or liposomal amphotericin B. Unfortunately, the availability of these medications is often inadequate in resource-limited settings. In this case, surgical debridement was successful but itraconazole was the only available anti-fungal with activity against *Mucorales*. Although the patient follow up was truncated there was a favorable in-hospital clinical response to itraconazole, which is in keeping with other reports of successful treatment with itraconazole monotherapy [44, 45].

The oral ulceration differential diagnosis includes squamous cell carcinoma, deep fungal infection, tertiary syphilis and acute necrotizing ulcerative gingivitis [46–49]. In our case, the acute course of disease goes against a diagnosis of squamous cell cancer. Negative non-treponemal syphilis serology and an atypical presentation exclude tertiary syphilis. The sinus involvement and presence of fungal hyphae makes acute necrotizing gingivitis unlikely. Most cases of rhinocerebral mucormycosis involve both sides of the palate, however hemi-palatal involvement has been reported [38, 39]. This patient presented with typical symptoms of rhinomaxillary mucormycosis including fever, headache, lethargy, pain and swelling of the maxillary sinus. Microscopy differentiated *Mucor* from aspergillus as broad, aseptate hyphae at 90° angles were observed.

This case illustrates that both prolonged haemolysis and recurrent fever occurring after parasite clearance in falciparum malaria are challenging to diagnose. A systematic approach to the differential diagnoses is vital as management can be critically different. The potential diagnosis of ceftriaxone-induced haemolytic anaemia is important as this drug is widely used empirically. Although the incidence of ceftriaxone-induced haemolytic anaemia is low, this diagnosis should be considered and ceftriaxone discontinued to prevent unnecessary blood transfusions, steroid treatment and mortality [30, 33]. Steroids are not recommended as treatment for severe malaria because steroids increase gastrointestinal bleeding, convulsions, and prolong coma [50, 51]. Investigating fever in resource-limited setting is challenging, as microbiologic diagnoses are frequently not possible. However, delayed evaluation of recurrent fever after parasite clearance may lead to unnecessary empirical anti-malarial and anti-bacterial use, which has important consequences on the individual and global level. Although invasive fungal infections rarely complicate severe malaria, early diagnosis and treatment with surgical debridement plus anti-fungals are essential to improve patient outcomes. Empiric overuse of antimicrobials contribute to anti-microbial resistant organisms, which is a global public health concern in this era of reduced research and development into novel antimicrobial therapeutics. To combat this pressing issue, improved microbial diagnostics are urgently needed in resource-limited settings.

## Learning points

- Potentially reversible drug-induced haemolytic anaemia may rarely complicate severe malaria following parasite clearance but needs to be considered to avoid unnecessary blood transfusion or steroid treatment.
- Ceftriaxone is a commonly used empiric antibiotic, however rarely (<0.1 % incidence) it may cause life threatening haemolysis. This diagnosis must be considered early as stopping the drug may be the only treatment needed and the haematologic response can be rapid.
- Caution must be exercised when considering steroid use in severe falciparum malaria, since no benefit has been shown, whereas there are potential adverse effects, such as in the present case where it may contribute to immunosuppression facilitating fungal infection.
- Recurrent fever after parasite clearance should prompt a thorough septic evaluation to avoid delays in diagnosis and overuse of empiric antimicrobials.
- Invasive fungal infections are a rare complication (<0.1 % incidence) of severe malaria but should be considered in individuals with prolonged fever after parasite clearance.
- Mucormycosis infection is a medical emergency requiring urgent surgical and medical management and carries high morbidity and mortality.

## Consent

Written informed consent was obtained from the patient for publication of this Case report and any accompanying images. A copy of the written consent is available for review by the Editor of this journal.

## Abbreviations

RBC: red blood cell
GCS: Glasgow coma score
BP: blood pressure
HR: heart rate
RR: respiratory rate
SaO_2_: oxygen saturation
AL: artemether lumefantrine combination
DAT: direct antiglobulin test
IAT: indirect antiglobulin test
DIIHA: drug-induced immune haemolytic anaemia
